# Health variations among breast-cancer patients from different disease states: evidence from China

**DOI:** 10.1186/s12913-020-05872-5

**Published:** 2020-11-11

**Authors:** Qing Yang, Xuexin Yu, Wei Zhang

**Affiliations:** 1 Institute of Hospital Management, West China Hospital, Sichuan University, 37 Guo Xue Alley, Chengdu, 610040 Sichuan China; 2 West China Biomedical Big Data Center, West China Hospital, Sichuan University, 37 Guo Xue Alley, Chengdu, 610040 Sichuan China

**Keywords:** Breast cancer, Disease states, Quality of life, Health utility, Mapping function

## Abstract

**Background:**

This study aimed to obtain health utility parameters among Chinese breast cancer patients in different disease states for subsequent health economics model. In addition, we aimed to explore the feasibility of establishing a breast cancer health utility mapping model in China.

**Methods:**

Multiple patient-reported health attributes were assessed, including quality of life, which was measured by the Functional Assessment of Cancer Therapy-Breast (FACT-B) instrument; health utility and self-rated health, which were measured by the EuroQol-5 Dimension-5 Level (EQ-5D-5L) questionnaire. Multivariate regression models, including a linear regression model, an ordinal logistic regression model and a Tobit model, were employed to analyze health differences among 446 breast cancer patients. Subgroup analyses were performed to examine differences in multiple dimensions of health derived from the FACT-B and EQ-5D-5L instruments. A mapping function was used to estimate health utility from quality of life. Rank correlation analyses were employed to examine the correlation between estimated and observed health utility values.

**Results:**

A total of 446 breast cancer patients with different disease states were analyzed. The health utility values of breast cancer patients in the P state (without cancer recurrence and metastasis), R state (with cancer recurrence within a year), S state (with primary and recurrent breast cancer for the second year and above), and M state (metastatic cancer) were 0.81 (SD ± 0.23), 0.90 (SD ± 0.12), 0.78 (SD ± 0.31), and 0.74 (SD ± 0.27), respectively. There were positive correlations between all scores, including every domain of the FACT-B instrument (*p* < 0.001). Results from multivariate analysis suggested that patients in the R and M states had lower scores for overall quality of life (R, β = − 9.45, *p* < 0.01; M, β = − 6.72, *p* < 0.05). Patients in the M state had lower health utility values than patients in the P state (β = − 0.11, *p* < 0.05). Estimated health utility values, which were derived from quality of life by using a mapping function, were significantly correlated with directly measured health utility values (*p* < 0.001).

**Conclusions:**

We obtained the health utility and health-related quality of life (HRQoL) scores of Chinese breast cancer patients in different disease states. Mapping health utility values from quality of life using four disease states could be feasible in health economic modelling, but the mapping function may need further revision.

## Background

As a leading cause of death among women, breast cancer accounts for approximately 23% of global cancer-related deaths [1]. Breast cancer is also one of the most common malignancies in China. According to data from the National Cancer Registry Annual Report 2018 [2], the number of women with breast cancer in China in 2014 was approximately 279,000, with an incidence rate of 41.82 per 100,000. In China, the number of breast cancer patients has escalated in both urban and rural areas in recent decades, leading to a drastic increase in health expenditures and disease burden for both society and patients’ families [3].

Understanding how to reduce the burden of disease and rationally allocate the limited health resources often requires health economic modelling. Recently, health economic modelling has gained an increased amount of attention in cancer research. Cost-utility analysis is an important method of health economic modelling. It aims to compare participants’ health attributes, such as quality-adjusted life years (QALYs), which incorporates the duration and health utility weights for specific health status [4]. The EQ-5D questionnaire, one of the most popular tools for measuring health utility, was developed by the European Quality of Life Organization and recommended as a generalized scale by the National Institute for Health and Care Excellence (NICE) in the UK [5]. Modelling research on health utility among breast cancer patients, however, is rare in developing countries, such as China, thus limiting the potential to conduct cost-utility analysis (CUA) in these regions [6].

In CUA, it is important to assess the health utility of different disease states to use utility in the state transition model. The International Society for Pharmacoeconomics and Outcomes Research (ISPOR) and the Society of Medical Decision Making (MDM) have published reports on best practices for state transition models (Markov and micro-simulation models) [7]. The Markov model is the most commonly used model for evaluating the health economics of breast cancer patients using utility values. Currently, the commonly used Markov model considers four different states of breast cancer [8, 9], which are based on the trajectory and economic modelling of breast cancer. The four mutually exclusive disease states for breast cancer patients include P, S, R, and M [10]. Patients in the P state are diagnosed with breast cancer within a year and do not suffer from cancer recurrence and metastasis. Patients in state S have primary and recurrent breast cancer for the second year and above. Patients in the R state have recurrent breast cancer for the first year. Finally, patients in the M state suffer from cancer metastasis. Patients in the P, R and M states generally receive various clinical treatments, such as surgery, chemotherapy and radiotherapy. Patients with cancer recurrence (R state) and metastasis (M state) may suffer more than patients in the P state since patients in these states may receive more treatments to cure the disease. Additionally, although cancer recurrence occurs among patients in the S state, they may generally report better health than patients in the R states, since patients in the former group might complete the treatment and return to normal life.

Although extensive observations have focused on health variations among breast cancer patients, especially as a function of various treatment approaches [11, 12], TNM stage [13], and social determinants such as race [14], marital status [13, 15], income [16], medical insurance [17], and education [13, 16], few investigations have directed attention to health variations based on disease states [10]. To the best of our knowledge, the study conducted by Lidgren et al. [10] was the only one that investigated health variations among breast cancer patients from four different states. Their descriptive study, however, provided little information about health variations after controlling for socioeconomic, demographic, and clinical attributes. These factors may significantly impact patients’ health regardless of the disease state. Furthermore, when measuring health utility, Lidgren et al. [10] employed a preference-based EQ-5D questionnaire to assess five dimensions of health with three-level severity. In contrast, the present study employed the refined version of the EQ-5D questionnaire, i.e., the EQ-5D-5L, which uses five-level severity to more accurately measure health utility.

At present, few studies have measured the health utility value of breast cancer in China [6]. Instead of health utility derived from preference scales, studies commonly assess quality of life by using numerous generalized or specific-preference scales, such as the FACT-B instrument and the quality of life instruments for cancer patients-breast cancer (QLICP-BR) [12, 15, 18] To transform quality of life into health utility, a mapping function is necessary [18]. Relevant research based on China’s population is, however, limited. It is unclear whether the mapping functions based on other Asian nations, such as Singapore, could be applicable to China’s population.

To offer guidance for data analyses and the interpretation of results, we offered two hypotheses here. First, we hypothesized that health variations might exist among breast cancer patients in different disease states even when socioeconomic, demographic, and clinical attributes were introduced (H_1a_). Specifically, patients with metastatic cancer (M state) and those with cancer recurrence (R state) may report worse health, particularly mental health, and feel more painful because they have a greater psychological burden and more severe adverse effects [19]. Additionally, we hypothesized that the variations would not exist in every dimension of health, since breast cancer patients generally do not suffer greatly in dimensions such as functional status, mobility, self-care, and usual activities (H_1b_). Second, we hypothesized that there would be a correlation between FACT-B and EQ-5D-5L scores, and we could use the published mapping function from the FACT-B instrument to the EQ-5D-5L questionnaire to obtain health utility parameters (H_2_).

To validate the hypotheses, this study analyzed health variations among patients in four disease states as a function of clinical, demographic, and socioeconomic attributes (H_1a+_H_1b_). In addition, we estimated health utilities for patients in four disease states by using a mapping algorithm developed by previous researchers [20] and compared estimated health utilities with measured health utilities to evaluate the feasibility of the existing mapping function in the subsequent health economic modelling across four disease states (H_2_). For a comprehensive assessment, this study measured the preference-based generic HRQoL, the health utility, by using the EuroQol-5 Dimension-5 Level (EQ-5D-5L) questionnaire. The EQ visual analogue scale (EQ-VAS) of the EQ-5D-5L questionnaire and the nonpreferred disease-specific FACT-B instrument were also administered.

## Methods

### Data source

We recruited both breast cancer outpatients and inpatients from Sichuan Oncology Hospital from November 2017 to May 2018. Ethical permission was granted by the Ethics Committee, West China School of Medicine/West China Hospital, Sichuan University (approval number 2017–255). We obtained permission to use the FACT-B instrument (Simplified Chinese version) and the EQ-5D-5L questionnaire (Simplified Chinese version).

### Study participants

The inclusion criteria were as follows. First, participants were clinically and/or pathologically diagnosed with breast cancer. Second, patients were aged 18 and above. Third, patients did not have any mental problems and had the ability to express. In addition, patients agreed to participate in this study. Informed consent was obtained from all participants. We excluded patients who had comorbidities, such as cardiovascular disease and mental health problems.

To achieve sufficient power, we calculated the sample size as follows:

$$ \mathrm{N}={\left(\frac{{\mathrm{t}}_{\upalpha}\hat{\upsigma}}{\updelta}\right)}^2 $$

We assumed that the probability of a type І error was 0.05. As suggested by prior research [21], the standard deviation of the health utility value of breast cancer patients was 0.16, the 95% confidence interval of the mean was from 0.82 to 0.85, and δ was 1/2 of the width of the confidence interval. Hence, the final target sample size was 440. We recruited 451 breast cancer patients, and five respondents did not complete the survey. Therefore, 446 participants were included in the data analyses.

### Measures

We measured participants’ quality of life by using the FACT-B instrument, which assesses quality of life across five dimensions: physiological well-being (PWB), social and family support (SWB), emotional well-being (EWB), functional well-being (FWB), and additional breast cancer symptoms (BCS). The FACT-B instrument consists of 37 questions. Since the scores of these five dimensions differed, we standardized them into a scale ranging from 0 to 100. The validity and reliability of the Chinese version of the FACT-B instrument were examined by prior investigators [22].

Additionally, patients’ health utility was measured by the EQ-5D-5L questionnaire (Simplified Chinese version). The EQ-5D-5L questionnaire assesses five health dimensions (mobility, self-care, usual activities, pain/discomfort, and anxiety/depression) with five levels of severity (no problems, slight problems, moderate problems, severe problems, and extreme problems/unable). The severity of each dimension was coded from 0 to 4, with 0 as the reference group. For example, a score of 0 for mobility represents that individuals have no problems with walking, and a score of 4 represents individuals who cannot walk. In addition, participants were required to report their self-rated health status on a scale ranging from 0 to 100, with 0 representing the worst health status and 100 representing the best health status one can imagine (EQ-VAS). The validity and reliability of the EQ-5D-5L questionnaire (Simplified Chinese version) were examined by previous researchers [23]. We calculated health utility by employing a value set based on Chinese data [24].

The main independent variable of interest is disease states, i.e., P, R, S, and M. In addition, we introduced covariates including TNM stage (0, I, II, III, and IV), surgical approaches (breast-conserving surgery, modified radical surgery vs. no surgery), menopause state (yes vs. no), radiotherapy (yes vs. no), chemotherapy (yes vs. no), targeted therapy (yes vs. no), endocrine therapy (yes vs. no), and inpatients (vs. outpatients) to control for clinical confounders that may affect patients’ health via adverse effects and are unrelated to disease states [12, 19].

Furthermore, we assessed patients’ demographic attributes (age and marital status) and socioeconomic characteristics, including educational attainment, household income, residence (urban vs. rural), occupation, and medical insurance type; these covariates were measured to control for the effect of social deprivation on health [15, 17].

### Data analysis

#### Data analysis methods for H_1_

To assess the differences in variables between the four disease states, descriptive analyses (Chi-square test, Fisher’s exact test, and ANOVA) were performed depending on the characteristics of the variables. We calculated health utilities by a value set developed based on previous research in China [24]. To assess the degree of overlap between instruments, Spearman’s rank correlation coefficient was calculated not only between each instrument but also between the domains of the FACT-B instrument.

ANOVA and the Wilcoxon rank-sum test were used to compare quality of life scores and health utility scores between different disease states.

Univariate analyses were conducted to determine potential predictors of participants’ health, which was reflected as overall scores on the FACT-B instrument, scores of each dimension of the FACT-B instrument, self-rated health (EQ-VAS), health utility (total scores of the EQ-5D-5L questionnaire), and the scores of every dimension of the EQ-5D-5L questionnaire. The univariate analyses included 15 independent variables, such as age, marital status, education attainment, residence, medical insurance, occupation, and household income. Independent variables with a *p*-value of less than 0.05 in the univariate analyses were then introduced in the multivariate analyses. Variance inflation factors were calculated to examine multicollinearity among independent variables in the modelling analyses.

We performed multiple regression models according to the distribution of the dependent variables. A linear regression model was performed for the overall FACT-B scores since the data were normally distributed. Ordinal logistic regression models were performed for BCS, FWB, EWB, SWB, PWB, and self-rated health, as the distributions of these variables were highly skewed. We also performed ordinal logistic regression analyses for the degree of mobility, self-care, usual activities, pain, and depression, as these variables were ordinal. For BCS, FWB, EWB, SWB, and PWB, we divided each variable into four balanced groups coded as 0, 1, 2, or 3, with 0 representing the lowest group and the reference group in the model; each group consisted of a similar number of participants. A similar process was used for self-rated health status, with five balanced groups coded as 0, 1, 2, 3, or 4, with 0 representing the group with the worst health. The Tobit model was performed for health utilities, as these data were right-censored.

#### Data analysis methods for H_2_

We analyzed the correlation between quality of life and health utilities from the EQ-5D-5L questionnaire by employing a rank correlation test. We estimated health utilities from the quality of life (assessed by the FACT-B instrument) by employing a mapping function derived from the Singaporean population [20]. We conducted a rank correlation test between estimated health utilities and those directly measured from participants using the EQ-5D-5L questionnaire.

The mapping function based on Singaporean patients was as follows [20]:

$$ \mathrm{Estimated}\ \mathrm{health}\ \mathrm{utility}=0.2846+0.0121\times \mathrm{PWB}+0.0044\times \mathrm{FWB}+0.0034\times \mathrm{BCS} $$

Data analyses were performed with SPSS 23.0. and SAS University Edition. A *p-*value of less than 0.05 was considered statistically significant.

## Results

### Descriptive analysis

Table 1 shows the sociodemographic and clinical characteristics of patients stratified by breast cancer disease status. Patients in the R state had the lowest average age (49.9 ± 7.08). A total of 84.3% of the participants in this study were outpatients. Specifically, 100% of patients in the S state, 90% in the R state, 79.07% in the M state, and 52.8% in the P state were outpatients (*p* < 0.001). Additionally, there was a significant difference in the number of patients who underwent modified radical surgery (74.22%) and the number of patients who underwent breast-conserving surgery (22.65%) or no surgery (3.14%). Specifically, the proportions of patients in the R state (80.00%) and M state (79.07%) who underwent modified radical surgery were greater than those of patients in the P state (66.40%) and S state (76.74%). A higher proportion of patients in the P state (24.8%) underwent breast-conserving surgery than those in the S (22.48%), R (20.00%), and M (18.60%) states. Furthermore, a greater proportion of patients in the M and R states underwent radiotherapy and endocrine therapy compared with those in the P and S states (*P* < 0.001). Menopause status appeared to vary across disease states. Patients’ socioeconomic attributes, such as household income and medical insurance type, also differed across disease states.

**Table 1:** Baseline characteristics of patients stratified by breast cancer disease status, (%)

	All (*N* = 446)	P (*N* = 125)	S (*N* = 258)	R (*N* = 20)	M (*N* = 43)	*p*
Age^a^	52.03(±8.97)	51.37(±8.62)	52.65(±8.75)	49.9(±7.08)	51.23(±11.6)	
Marital status						
Married	92.15	6.40	6.59	10.00	18.60	
Others	7.85	6.40	6.59	10.00	18.60	
Educational attainment						
Illiteracy and primary school	32.29	39.20	31.40	35.00	16.28	
Junior high school	33.63	26.40	35.66	35.00	41.86	
Senior high school	19.73	19.20	19.38	20.00	23.26	
College or above	14.35	15.20	13.57	10.00	18.60	
Hukou system						
Rural	49.33	56.00	47.67	50.00	39.53	
Urban	50.67	44.00	52.33	50.00	60.47	
Medical insurance						
New rural cooperative scheme and others	44.62	56.00	39.53	55.00	37.21	*
Urban employees	43.27	36.80	44.96	40.00	53.49	
Urban residents	12.11	7.20	15.50	5.00	9.30	
Occupation						
Unemployed	33.18	30.40	35.66	50.00	18.60	
Retired	21.52	16.00	23.64	10.00	30.23	
Blue-collar	27.58	33.60	24.03	35.00	27.91	
White-collar	17.71	20.00	16.67	5.00	23.26	
Household income						
< 30,000 RMB	52.47	53.60	50.39	90.00	44.19	**
30,000–80,000 RMB	33.18	26.40	37.21	10.00	39.53	
> 80,000 RMB	14.35	20.00	12.40	0.00	16.28	
TNM stage						
0 and I	19.95	25.60	20.54	0.00	9.30	***
II	50.22	44.80	58.14	50.00	18.60	
III	22.20	25.60	20.93	20.00	20.93	
IV	7.62	4.00	0.39	30.00	51.16	
Inpatients (vs. outpatients)						
Outpatients	84.30	52.80	100.00	90.00	79.07	***
Inpatients	15.70	47.20	0.00	10.00	20.93	
Surgery						
No surgery	3.14	8.80	0.78	0.00	2.33	*
Breast-conserving surgery	22.65	24.80	22.48	20.00	18.60	
Modified radical surgery	74.22	66.40	76.74	80.00	79.07	
Radiotherapy (yes vs. no)	60.76	44.00	63.95	95.00	74.42	***
Chemotherapy (yes vs. no)	91.70	88.80	92.25	100.00	93.02	
Targeted therapy (yes vs. no)	9.64	12.00	8.14	0.00	16.28	
Endocrine therapy (yes vs. no)	68.83	38.40	81.01	100.00	69.77	***
Menopause (yes vs. no)	85.87	77.60	90.31	90.00	81.40	*

^***^p < .001; ^**^p < .01; ^*^p < .05

^a^represents mean (±standard deviation)

**Fig. 1:**
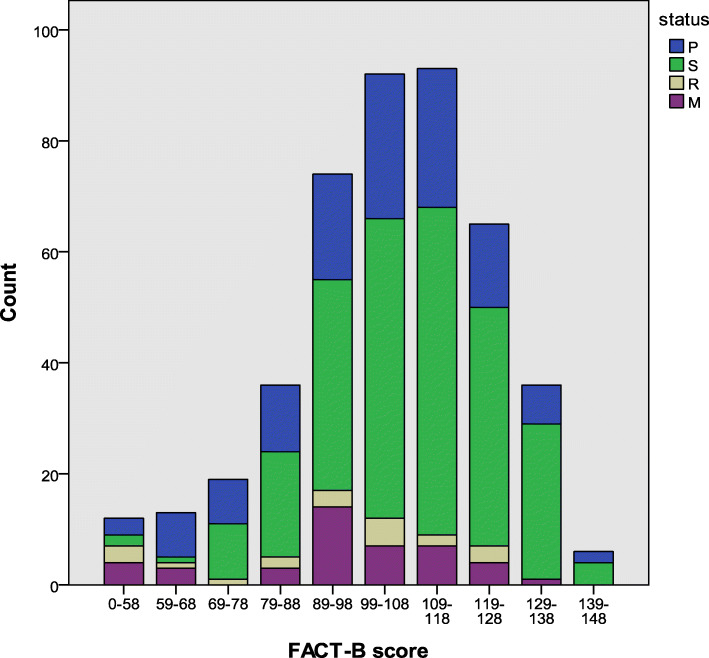
Distribution of the FACT-B scores

**Fig. 2:**
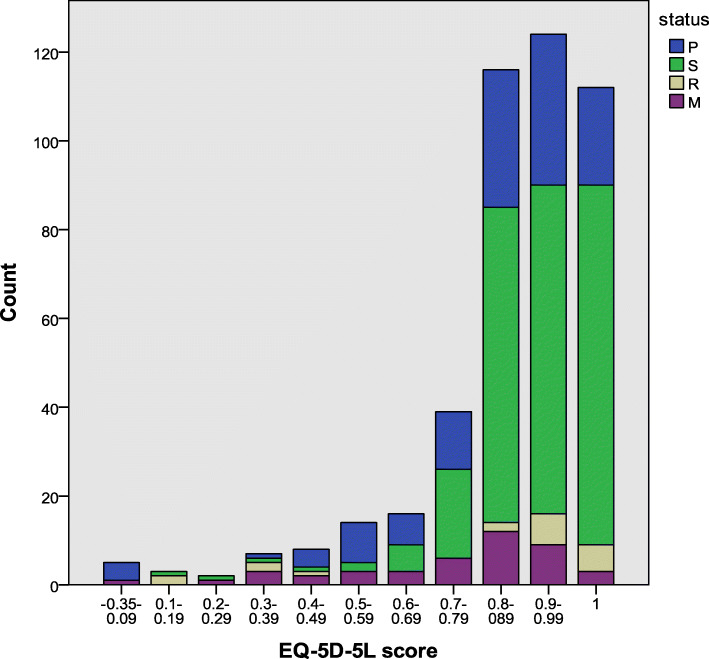
Distribution of the EQ-5D-5L scores

**Fig. 3:**
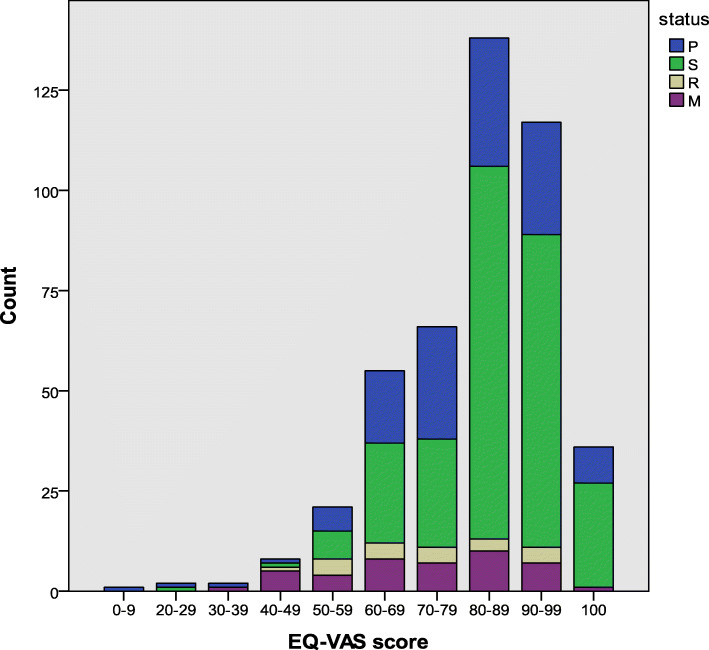
Distribution of the EQ-VAS scores

Table 2 presents the quality of life stratified by disease states, including HRQoL, EQ-5D-5L, and EQ-VAS scores. The median (IQR) utility of breast cancer patients in the P, R, S and M states was 0.89 (0.73–0.95), 0.94 (0.86–1.00), 0.92 (0.65–1.00), and 0.85 (0.63–0.90), respectively. To reflect the different quality of life scores and health utility scores across the four disease states, the FACT-B, EQ-5D-5L and EQ-VAS scores were used as the x-axis to draw histograms. The histograms representing the distributions of these scores are shown in Figs. 1, 2 and 3. A strong ceiling effect was observed for the EQ-D-5 L score. According to the descriptive analysis, patients in the S state appeared to have better health in all dimensions (Table 2). Patients in the M state had the worst health as indicated by the EWB, EQ-5D-5L, and EQ-VAS scores, while patients in the R state reported the lowest scores for overall quality of life, as indicated by the FACT-B instrument, and the EQ-VAS.

**Table 2:** Health utility and HRQoL scores by disease states, median (IQR)

Variable	All (*N* = 446)	P (*N* = 125)	S (*N* = 258)	R (*N* = 20)	M (*N* = 43)	*p*
FACT-B^a^	70.29(±13.33)	68.09 (±13.85)	73.03 (±11.68)	61.66 (±16.86)	64.27 (±14.84)	***
PWB	85.71 (75.00–92.86)	82.14 (71.43–89.29)	85.71 (78.57–96.43)	78.57 (71.43–91.07)	82.14 (67.86–85.71)	***
SWB	67.86 (57.14–85.71)	67.86 (57.14–85.71)	71.43 (57.14–85.71)	55.36 (39.29–71.43)	67.86 (50.00–78.57)	*
EWB	79.17 (62.50–91.67)	79.17 (58.33–91.67)	83.33 (70.83–91.67)	62.50 (45.83–85.42)	62.50 (50.00–79.17)	***
FWB	57.14 (42.86–71.43)	53.57 (42.86–67.86)	60.71 (46.43–75.00)	48.21 (32.14–53.57)	50.00 (42.86–64.29)	***
BCS	72.50 (62.50–77.50)	70.00 (60.00–77.50)	72.50 (65.00–80.88)	67.50 (53.75–80.00)	70.00 (57.50–80.00)	*
EQ-5D-5L	0.90 (0.83–1.00)	0.89 (0.73–0.95)	0.94 (0.86–1.00)	0.92 (0.65–1.00)	0.85 (0.63–0.90)	***
EQ-VAS	80.00 (70.00–90.00)	80.00 (70.00–90.00)	80.00 (80.00–90.00)	70.00 (55.00–82.50)	70.00 (60.00–80.00)	***

^***^p < .001; ^**^p < .01; ^*^p < .05

^a^represents mean (±standard deviation)

### Variations in quality of life

Table 3 shows the Spearman’s rank correlation coefficients between the scores. There were positive correlations between all scores, including the individual domains of the FACT-B instrument. The correlation coefficient between the FACT-B and EQ-5D-5L scores was higher than that between the FACT-B and EQ-VAS scores.

**Table 3:** Spearman’s rank correlation coefficients between the scores (coefficients)

	FACT-B						EQ-VAS
	Total	PWB	SWB	EWB	FWB	BCS	
EQ-5D-5L	0.642^***^	0.601^***^	0.389^***^	0.558^***^	0.471^***^	0.545^***^	0.442^***^
FACT-B		0.657^***^	0.741^***^	0.799^***^	0.752^***^	0.744^***^	0.466^***^
PWB			0.298^***^	0.575^***^	0.330^***^	0.543^***^	0.375^***^
SWB				0.458^***^	0.574^***^	0.389^***^	0.231^***^
EWB					0.481^***^	0.605^***^	0.445^***^
FWB						0.379^***^	0.467^***^
BCS							0.283^***^

^***^p < .001; ^**^p < .01; ^*^p < .05

Consistent with the descriptive analysis, the results from multivariate analysis suggested that patients in the R and M states had lower scores for overall quality of life (R,β = − 9.45, *p* < 0.01; M, β = − 6.72, *p* < 0.05) after adjusting for other covariates in the model (Table 4). Additionally, patients in the R state had lower FWB (β = − 0.98, *p* < 0.05) and SWB scores (β = − 1.41, *p* < 0.01). Patients in the M state appeared to have lower EWB (β = − 1.07, *p* < 0.05) and SWB scores (β = − 0.82, *p* < 0.05).

**Table 4:** Multivariate analyses of variations in quality of life derived from FACT-B (coefficients)

Independent variable	Quality of life	BCS	FWB	EWB	SWB	PWB
Age (in a unit of years)					−0.01	
Married (vs. others)					0.74^*^	
Educational attainment						
Illiteracy and primary school	Ref.	Ref.	Ref.		Ref.	Ref.
Junior high school	3.09^*^	0.48^*^	0.25		0.79^**^	0.22
Senior high school	4.24^*^	0.56^*^	0.33		0.65^*^	0.25
College or above	2.80	−0.23	0.45		0.84^*^	0.18
Urban (vs. rural)			− 0.03			
Medical insurance						
New rural cooperative scheme and others	Ref.	Ref.	Ref.	Ref.		Ref.
Urban employees	1.58	−0.01	0.09	0.33		0.51^*^
Urban residents	4.58^*^	0.51	0.23	0.61^*^		0.98^**^
Occupation						
Unemployed			Ref.			
Retired			−0.12			
Blue-collar			−0.54			
White-collar			−0.23			
Household income						
< 30,000 RMB			Ref.		Ref.	
30,000–80,000 RMB			0.03		−0.36	
> 80,000 RMB			0.87**		0.22	
Inpatients (vs. outpatients)	−9.25^***^	−0.48	−0.46	− 0.94^**^	− 0.74^*^	−1.50^***^
TNM stage						
0 and I	Ref.		Ref.	Ref.	Ref.	
II	−2.59		−0.29	0.05	− 0.28	
III	−3.26		−0.29	0.01	−0.27	
IV	−3.63		0.29	−0.45	0.12	
Surgery						
No surgery			Ref.			
Breast-conserving surgery			0.66			
Modified radical surgery			0.27			
Radiotherapy (yes vs. no)	−9.25^***^					0.32
Targeted therapy (yes vs. no)				0.46		
Endocrine therapy (yes vs. no)	−0.52					0.02
State						
P	Ref.	Ref.	Ref.	Ref.	Ref.	Ref.
S	−0.16	0.21	0.30	− 0.11	− 0.29	0.17
R	−9.45^**^	−0.07	−0.98^*^	− 0.91	−1.41^**^	−0.67
M	−6.72^*^	−0.05	−0.59	−1.07^*^	− 0.82^*^	−0.46

^***^p < .001; ^**^p < .01; ^*^p < .05

Variables without coefficients did not make statistically significant difference in univariate analyses (results not presented here); therefore, they were not introduced into multivariate analyses

The results of the multivariate analysis differed from those of the descriptive analysis in terms of differences in BCS and PWB between disease states (Tables 1 and 2). There appeared to be no differences in BCS and PWB across disease states after controlling for other covariates.

### Variations in health utility

There were differences in health utility, depression, and pain between disease states (Table 5). Specifically, patients in the M state had lower health utility values (β = − 0.11, *p* < 0.05), higher levels of pain (β = 1.17, *p* < 0.01) and higher levels of depression (β = 1.21, *p* < 0.01) than patients in the P state. In contrast, there appeared to be no differences in the other dimensions of health utility, including mobility, self-care, and usual activities, between patients in the M state and those in the P state.

**Table 5:** Multivariate analyses of variations in health utility from EQ-5D-5L (coefficients)

Independent variable	Items of EQ-5D-5L					EQ-5D-5L	EQ-VAS
	Mobility	Self-Care	Usual activities	Pain	Depression		
Age (in a unit of years)	0.03						
Married (vs. others)	−0.89^*^	− 0.87^*^	− 0.73			0.06	
Educational attainment							
Illiteracy and primary school	Ref.	Ref.	Ref.				
Junior high school	0.07	−0.06	−0.45				
Senior high school	−0.32	0.02	−0.32				
College or above	0.40	−0.46	−0.52				
Urban (vs. Rural)	−0.53		−1.58^*^				0.67
Medical insurance							
New rural cooperative scheme and others	Ref.	Ref.	Ref.	Ref.	Ref.	Ref.	Ref.
Urban employees	−0.52	−0.30	1.08	− 0.43^*^	−0.46^*^	0.05^*^	−0.16
Urban residents	−0.91	−0.51	− 0.79^*^	−0.72^*^	− 0.77^*^	0.09^*^	0.62^*^
Occupation							
Unemployed	Ref.	Ref.					
Retired	0.59	0.23					
Blue-collar	0.47	0.37					
White-collar	−0.11	−0.42					
Household income							
< 30,000 RMB			Ref.				
30,000–80,000 RMB			−0.31				
> 80,000RMB			−0.01				
Inpatients (vs. outpatients)	2.05^***^	1.41^**^	1.53^***^	1.30^***^	0.95^**^	−0.22^***^	−0.47
TNM stage							
0 and I	Ref.	Ref.	Ref.	Ref.	Ref.	Ref.	Ref.
II	0.63	0.23	0.70^*^	0.19	0.11	−0.03	0.09
III	0.45	0.72	0.90^*^	0.31	−0.33	−0.03	− 0.14
IV	1.37^*^	0.60	1.20^*^	1.10^*^	0.18	−0.10	−0.12
Surgery							
No surgery	Ref.						Ref.
Breast-conserving surgery	0.45						0.67
Modified radical surgery	0.86						0.24
Radiotherapy (yes vs. no)	0.00					0.01	
Targeted therapy (yes vs. no)							1.04^**^
Endocrine therapy (yes vs. no)		−0.03	0.44			−0.01	
Chemotherapy (yes vs. no)		− 0.56	−0.77^*^				
Menopause (yes vs. no)		−0.21					
State							
P	Ref.	Ref.	Ref.	Ref.	Ref.	Ref.	Ref.
S	0.22	−0.53	−0.39	0.31	− 0.11	0.00	0.35
R	0.44	−0.04	0.32	0.24	0.56	−0.07	−1.12^*^
M	0.30	0.44	0.27	1.21^**^	1.17^**^	−0.11^*^	− 0.79

^***^p < .001; ^**^p < .01; ^*^p < .05

Variables without coefficients did not make a statistically significant difference in univariate analyses (results not presented here); therefore, they were not introduced into multivariate analyses

### Differences in self-rated health

Similar to the results from the descriptive analysis (Table 2), the multivariate analysis results suggested that there were differences in self-rated health between patients in the R state and those in the P, S, and M states (Table 5). Compared with patients in the P state, patients in the R state had lower self-rated health even after controlling for all other covariates in the model (β = − 1.12, *p* < 0.05). There was no significant difference in self-rated health for patients in the other three states (Table 5).

The above results supported our first hypothesis and indicated that patients in different disease states might have different health statuses, while when sociodemographic and clinical attributes were considered, some differences disappeared.

### The feasibility of using four disease states in health economic modelling

The results from the rank correlation analysis suggested that overall scores of quality of life were significantly correlated with health utility values derived from the EQ-5D-5L questionnaire (Table 6). This correlation existed among patients in all disease states; however, the correlation coefficient for patients in the R state (*r* = 0.919*, p* < 0.001) was higher than that for patients in the other states.

**Table 6:** Correlation between health utility and estimated health utility

States	FACT-B	EQ-5D-5L	*r*^a^	Estimated health utility	*r*^b^
P	68.09 ± 13.85	0.81 ± 0.23	0.680^***^	0.70 ± 0.09	0.719^***^
S	73.03 ± 11.68	0.90 ± 0.12	0.568^***^	0.75 ± 0.06	0.602^***^
R	61.66 ± 16.86	0.78 ± 0.31	0.919^***^	0.69 ± 0.10	0.961^***^
M	64.27 ± 14.84	0.74 ± 0.27	0.720^***^	0.69 ± 0.09	0.715^***^
All	70.29 ± 13.33	0.86 ± 0.19	0.642^***^	0.73 ± 0.08	0.681^***^

^***^p < .001; ^**^p < .01; ^*^p < .05

*r*^a^ represents the correlation between patient-reported quality of life and health utility

*r*^b^ represents the correlation between patient-reported health utility and estimated health utility

In addition, estimated health utilities derived from quality of life measures were significantly correlated with health utilities directly measured from patients (Table 6). The results suggested that the mapping function generated more accurate health utilities for patients in the P, S, and R states than for those in the M state (*r* = 0.720 vs. *r* = 0.715) (H_2_).

## Discussion

Extensive observations have focused on breast cancer patients’ health while overlooking the variations in health across different disease states. Even fewer investigators have examined multiple dimensions of health. The utility parameters used in only a few studies in China come from studies in other countries [25, 26], so it is urgent to conduct a quality of life study based on the Chinese population and obtain health utility values. Furthermore, this study extended the research on mapping the function of health utility to China’s breast cancer patients, which have rarely been studied [20, 24].

This study has three major implications. First, health differed across different disease states, which may be due to different levels of psychological burden and adverse effects from treatment in M and R states. Consistent with prior Swedish observations [10], this study revealed that patients in the S state have higher health utility values. However, the mean value of the EQ-5D-3L questionnaire for patients in the S state was 0.779 in the Swedish study [10], which was slightly lower than that in our study. This difference may be caused by the different demographic characteristics of the research participants and the different dimensions of the EQ-5D questionnaire. However, the 5-dimensional EQ-5D questionnaire may be more accurate in measuring the health utility of breast cancer patients. Meanwhile, our study further revealed that after controlling for other covariates, there was no difference between patients in the S state and those in the P and R states, while patients in the M state had lower health utilities relative to patients in the other three states. Liu et al. [27] conducted studies on breast cancer in China and found that metastatic breast cancer had a lower health utility value than nonmetastatic breast cancer, but they did not report specific health utility values for different disease states. A South Korean study that used standard gambling to measure the health utility of breast cancer found that the health utility of metastatic cancer patients was lower than that of patients with local recurrence and other states [28]. The results from the subgroup analysis demonstrated that patients in the M state had higher odds of experiencing pain and depression, and they may have a poor quality of life, especially with respect to the family and social support and emotional well-being. The results suggested that extra care should be given to patients in the M state, as metastatic cancers often lead to greater adverse effects and higher levels of pain, which may decrease patients’ quality of life and health utility [19, 28, 29].

In addition, our results suggested that patients in the R state had the worst quality of life and self-rated health, which differed from variations in health utility. Although local recurrence requires similar treatment to the first treatment, the quality of life of patients in the R state is significantly lower than that of patients in the P state, mainly due to the patient’s response, and the patient’s fear of treatment, pain and discomfort may lead to a decrease in quality of life [10, 30, 31]^]^. The results of the study indicate that the results of different quality of life measurement tools are slightly different. However, the scores of the three scales and the EQ-5D-5L values obtained by FACT-B conversion using the mapping model were different across disease states, indicating that the three methods have good discrimination when measuring the quality of life of breast cancer patients and can be used for evaluating quality of life in breast cancer patients. Our results further demonstrated that patients in the R state had lower levels of functional well-being and social and family support than patients in the M, P and S states. The results suggested that patients experiencing cancer recurrence may have a heavier psychological burden.

The second implication is that there did not appear to be differences in all dimensions, including BCS, PWB, mobility, self-care and usual activities, between disease states. The results suggested that patients in different disease states reported similar scores for these health attributes after controlling for sociodemographic and clinical attributes, even though there appeared to be statistically significant differences in the univariate analyses.

Third, our study confirmed that the application of four disease states in health economic modelling holds great promise since patients in the four disease states reported significantly different health utilities. The results also revealed that the current mapping function derived from China’s population needs to be refined to accurately estimate health utilities from quality of life since the correlation coefficient decreased for patients in the M state. The FACT-B instrument is the most widely used specific quality of life tool in China, but as far as we know, a Singaporean study by Yin et al. [32, 33] was the only study to explore the feasibility of mapping FACT-B scores to the EQ-5D-5L questionnaire. This study was the first to explore the feasibility of mapping FACT-B scores to the EQ-5D-5L questionnaire in China. Using this mapping model to calculate the mapped utility value [32], it was found that the EQ-5D-5L utility value score was correlated with the FACT-B mapping utility value score (*p* < 0.05), thus indicating the feasibility of using different states. The correlation coefficient between the value scores was between 0.602 and 0.961. The utility value of each disease state after mapping was lower than the true value measured by the EQ-5D-5L questionnaire, and the difference between the FACT-B scores of patients in the R state and those in the M state became equal after the conversion to the health utility value. This may be related to the previous research being conducted in Singapore; the research objectives of that study were different from this study, and the EQ-5D-5L questionnaire is related to the UK and Japan’s integral utility system, suggesting that foreign mapping algorithms need to be localized to be used in the Chinese population.

This study presents three aspects of improvement over prior studies. First, the current study included breast cancer patients from four disease states and was thus more inclusive than prior investigations that generally focused on one or two states [11, 12, 29]. In particular, the health utility scores obtained in this study are useful for cost-utility analysis using QALYs as a metric. Second, this study included multiple dimensions of health and used three measurement tools, while the majority of existing examinations usually measured one health outcome [15]. Third, this study was the first to explore the feasibility of mapping the FACT-B instrument to the EQ-5D-5L questionnaire in China.

Despite the strengths, this study has several limitations. First, this study collected data from a single health center, which mitigated the representativeness of the results to China’s breast cancer patients. Second, the cross-sectional study design provided weak evidence for the robustness of the results and limited information for QALYs that incorporated the duration of each state. Third, when conducting the study, we considered whether the patient was an inpatient or an outpatient for further consultation, but we did not consider the specific length of hospitalization or time since discharge. For example, the quality of life of outpatients after discharge may vary at different times after treatment. In the future, we aim to carry out longitudinal research to describe the changes and differences in the quality of life of breast cancer patients over time and across disease states. Last, participants in this study were aged from 43 to 61 years old, thus restricting the external validity of our results to elderly and younger patients.

## Conclusions

We obtained the health utility, HRQoL and VAS scores of Chinese cancer patients in the P, S, R and M states measured by the EQ-5D-5L, EQ-VAS and FACT-B instruments. To the best of our knowledge, this is the first study to evaluate the quality of life of breast cancer patients across multiple disease states using a variety of measurement tools. In our study, all scores were correlated with one another. The quality of life of breast cancer patients differed across disease states. Early diagnosis, treatment and the reduction of recurrence and metastasis are beneficial for improving the quality of life of patients. We also use the published mapping model to verify the feasibility of mapping the FACT-B instrument to the EQ-5D-5L questionnaire in China. However, we suggest that in the future, it is better to establish a localized health utility mapping function in China. Our work will help to develop the use of cost-utility analysis in the Chinese environment as breast cancer treatments continue to increase.

## Data Availability

The dataset are available from the corresponding author on reasonable request.

## Abbreviations

BCS: Additional concerns for breast cancer
CUA: Cost-utility analysis
EQ-5D-5 L: EuroQol-5 Dimension-5 Level
EQ-VAS: EQ visual analogue scale
EWB: Emotional well-being
FACT-B: Functional Assessment of Cancer Therapy-Breast
FWB: Functional well-being
HRQoL: Health-related quality of life
ISPOR: International Society for Pharmacoeconomics and Outcomes Research
MDM: Medical Decision Making
NICE: National Institute for Health and Care Excellence
PWB: Physical well-being
QALYs: Quality-adjusted life years
QLICP-BR: Quality of life instruments for cancer patients-breast cancer
Ref: Reference
SWB: Social/family well-being
